# Precise evaluation of tissue culture-induced variation during optimisation of in vitro regeneration regime in barley

**DOI:** 10.1007/s11103-020-00973-5

**Published:** 2020-02-11

**Authors:** Renata Orłowska, Piotr Tomasz Bednarek

**Affiliations:** Department of Plant Physiology and Biochemistry, Plant Breeding and Acclimatization Institute–National Research Institute, Błonie, 05-870 Radzików, Poland

## Abstract

**Key message:**

The Taguchi method and metAFLP analysis were used to optimise barley regenerants towards maximum and minimum levels of tissue culture-induced variation. The subtle effects of symmetric and asymmetric methylation changes in regenerants were identified.

**Abstract:**

Plant tissue cultures (PTCs) provide researchers with unique materials that accelerate the development of new breeding cultivars and facilitate studies on off-type regenerants. The emerging variability of regenerants derived from PTCs may have both genetic and epigenetic origins, and may be desirable or degrade the value of regenerated plants. Thus, it is crucial to determine how the PTC variation level can be controlled. The easiest way to manipulate total tissue culture-induced variation (TTCIV) is to utilise appropriate stress factors and suitable medium components. This study describes the optimisation of in vitro tissue culture-induced variation in plant regenerants derived from barley anther culture, and maximizes and minimizes regenerant variation compared with the source explants. The approach relied on methylation amplified fragment length polymorphism (metAFLP)-derived TTCIV characteristics, which were evaluated in regenerants derived under distinct tissue culture conditions and analysed via Taguchi statistics. The factors that may trigger TTCIV included CuSO_4_, AgNO_3_ and the total time spent on the induction medium. The donor plants prepared for regeneration purposes had 5.75% and 2.01% polymorphic metAFLP loci with methylation and sequence changes, respectively. The level of TTCIV (as the sum of all metAFLP characteristics analyzed) identified in optimisation and verification experiments reached 7.51 and 10.46%, respectively. In the trial designed to produce a minimum number of differences between donor and regenerant plants, CuSO_4_ and AgNO_3_ were more crucial than time, which was not a significant factor. In the trial designed to produce a maximum number of differences between donor and regenerant plants, all factors had comparable impact on variation. The Taguchi method reduced the time required for experimental trials compared with a grid method and suggested that medium modifications were required to control regenerant variation. Finally, the effects of symmetric and asymmetric methylation changes on regenerants were identified using novel aspects of the metAFLP method developed for this analysis.

**Electronic supplementary material:**

The online version of this article (10.1007/s11103-020-00973-5) contains supplementary material, which is available to authorized users.

## Introduction

Plants regenerated via tissue culture methods of androgenesis or somatic embryogenesis are not completely uniform at morphological (Al Hattab et al. 2017), genetic (Hazubska-Przybył and Dering 2017) and epigenetic (Han et al. 2018) levels. In vitro plant regeneration from anthers or isolated microspores in barley (Bednarek et al. 2007), triticale (Machczyńska et al. 2015) and many other species (Fiuk et al. 2010) induce numerous changes that affect genome sequences and DNA methylation patterns. These genetic changes occur with varying frequency, from complete absence (Palombi and Damiano 2002; Tiwari et al. 2015) to 50% (Thomas et al. 2006) or 72% (Bairu et al. 2006). The epigenetic differences varied from 0.07% (Bobadilla Landey et al. 2013) to 35% (Gimenez et al. 2016) or 52% (Dann and Wilson 2011). The direction of (epi)mutations depended on the species; in triticale, genetic changes prevailed over methylation alterations (Machczyńska et al. 2014a), whereas the opposite was observed in barley (Bednarek et al. 2007). Regenerated barley plants displayed increased DNA methylation levels compared with donor plants (source of explants), whereas regenerated triticale plants displayed reduced DNA methylation within analysed restriction sites. Methylation changes were linked to the activation of transposons that might be responsible for genetic variation (Gimenez et al. 2016; Orłowska et al. 2016). Plants regenerated in vitro from somatic tissues also display numerous (epi)mutations (Bednarek and Orłowska 2019) as indicated in triticale (Machczyńska et al. 2014b), although those changes did not appear to affect plant regenerants at the morphological level (Machczyńska et al. 2014b, 2015). Conversely, some of the induced (epi)mutations were inherited during successive generative cycles. The alterations in DNA methylation patterns were species-specific. In barley, the DNA methylation level increased in regenerant plants and remained unchanged during a generative cycle (Orłowska et al. 2016). Reduced DNA methylation was observed in triticale regenerants, and it continued decreasing for the next few generative cycles before slightly increasing (Machczyńska et al. 2014a). Thus, tissue culture-induced variation at the molecular level can be transmitted to progeny (Rosato et al. 2016), and several generative cycles may or may not stabilise it (Machczyńska et al. 2014a; Orłowska et al. 2016). Further studies are needed to determine whether regenerant variation can be controlled. This knowledge will be crucial to continue exploiting tissue culture-based plant regeneration approaches.

Several approaches can be used to study in vitro tissue culture-induced variation in regenerated plants. Two of these approaches are based on amplified fragment length polymorphisms (AFLP) analysis. Methylation-sensitive amplified polymorphism (MSAP) (Baranek et al. 2016) uses HpaII and MspI endonuclease to study methylation changes within DNA sequences at the restriction sites. This approach was recently extended, enabling quantification of only the site DNA methylation alterations (Bednarek et al. 2017). Methylation amplified fragment length polymorphism (metAFLP) enables quantitative analysis of both sequence and DNA methylation changes affecting Acc65I–KpnI restriction sites (Bednarek et al. 2007; Chwedorzewska and Bednarek 2012; Machczyńska et al. 2014b; Wódkiewicz et al. 2018). Both methods were successfully used for the analysis of tissue culture-induced variation (Coronel et al. 2018; Gimenez et al. 2016; Goyali et al. 2018; Mikula et al. 2011). Reversed phase-high performance liquid chromatography (RP-HPLC) provides robust data on DNA methylation changes affecting the whole genome, but the method may not detect many subtle phenomena (Machczyńska et al. 2014a). Bisulfite sequencing was tested recently, and provided promising results on tissue culture-induced variation in *Boesenbergia rotunda* (Karim et al. 2018) and sugar beet (Zakrzewski et al. 2017). Diversity arrays technology sequencing methylation analysis (DArTseqMet, developed by Diversity Arrays Technology, https://www.diversityarrays.com/technology-and-resources/dartseq/) is a promising technology for methylation analysis, although it is relatively costly for routine analysis. Thus, it is reasonable to use either metAFLP or MSAP in combination with RP-HPLC for preliminary studies because these methods provide quantitative characteristics that might be useful for optimisation of in vitro tissue culture approaches towards maximum or minimum variation depending on specific needs.

The in vitro tissue culture-induced variation could be optimised either via split-plot designs (SPD) (Schmildt et al. 2015) using completely randomized designs (CRD) (Kumar et al. 2017) and randomized complete block designs (RCBD) (Amer et al. 2016), or via factorial designs such as the Taguchi method, a statistical approach used to optimise experimental parameters (Taguchi 1986). The SPD, CRD and RCBD methods require a large number of experiments, which are challenging to manage in tissue cultures due to the lack of sufficiently large numbers of explants (Nas et al. 2005). Nas et al. (2005) showed that methods based on fractional factorial designs, such as Plackett-Burman designs (PBD) or fractional factorial design (FFD), can be used in plant tissue culture experiments and significantly reduce the number of optimisation experiments compared with that needed for SPD, CRD and RCBD. The Taguchi method utilises the same concept with two-, three- and mixed-level fractional factorial designs, which renders this a robust method for optimisation of industrial processes (Rao et al. 2008; Ravanfar et al. 2015). The Taguchi method has been applied successfully in a range of biological experiments (Orłowska et al. 2020; Ramakrishna et al. 2013; Ravanfar et al. 2015).

The present study uses the Taguchi method to optimise the in vitro tissue culture-induced variation (genetic and DNA methylation) in barley regenerants towards minimum and maximum levels of changes compared with donor plants.

## Materials and methods

### Donor plant preparation and plant regeneration

The spring barley cultivar NAD2 was provided by Poznan Plant Breeders Ltd (Nagradowice, Poland). Barley plants were grown in pots (26 cm width × 23 cm height) filled with a mixture of soil and sand (3:1). A total of 24 plants were used with six plants per pot. Plants were grown in a growth chamber maintained under controlled conditions [16 h light/8 h dark photoperiod, 16 °C/12 °C during day/night, and approximately 190 µE m^−2^ s^−1^ light intensity using sodium lamps (light intensity was measured using the GLOptic spectroradiometer, Poznan, Poland)] until spikes were collected. During the vegetative stage, plants were fertilised (Florovit) every 2 weeks. Spikes were harvested when microspores were in the mid- to late-uninucleate stage, which was determined using acetic carmine staining. Suitable barley spikes were incubated in the dark in a pot containing water for 21 days at 4 °C. Subsequently, spikes were sterilised by soaking in 70% ethanol for 1 min, soaking in 10% sodium hypochlorite for 20 min and then washing four times with sterile distilled water (Oleszczuk et al. 2004). Anthers were placed under sterile conditions on species-specific solid induction medium [N_6_L containing macro- and microelements (Chu 1978)] supplemented with 2 mg l^−1^ 2,4-D (2,4-dichlorophenoxyacetic acid), 0.5 mg l^−1^ NAA (naphthaleneacetic acid), and 0.5 mg l^−1^ kinetin, and incubated in the dark at 26 °C until the end of callus formation. After 2–4 weeks of anther culture, the androgenic structures (1.5–3 mm in size) were transferred to regeneration medium K4NB (Kumlehn et al. 2006) supplemented with 0.225 mg l^−1^ BAP (6-benzylaminopurine). Calli and embryo-like structures were incubated at 26 °C under a 16 h light/8 h dark photoperiod (50 µE m^−2^ s^−1^ light intensity). Green regenerated plantlets were transferred to glass flasks containing N6I rooting medium (Chu 1978) supplemented with 2 mg l^−1^ indole-3-acetic acid (IAA). The developed plantlets were transferred to pots and grown in a greenhouse. The chromosome number doubled spontaneously. The ploidy of regenerants was evaluated based on a comparison of plant morphology (plant height, leaf shape) and fertility between maternal and regenerated plants. Spikes from randomly chosen regenerants were self-pollinated. Progenies derived from doubled haploid (DH) regenerants served as donors (D) of explants (anthers) for the optimisation (Experiment 1) and verification (Experiment 2) of plant regeneration via androgenesis.

#### Experiment 1

In vitro plant regeneration via anther culture was conducted using the protocol described above, which reflected control conditions (M1). Media with varying concentrations of CuSO_4_ and AgNO_3_ and varying time spans on induction medium (from plating anthers on induction medium to collecting calli and subsequent transfer to regeneration medium) were tested for their effects on androgenesis according to the Taguchi method. These requirements resulted in eight different trials (Table 1).

**Table 1:** Induction tests for in vitro plant regeneration of barley via androgenesis (anther cultures)

Trial	Factors		
	CuSO_4_ (µM)	AgNO_3_ (µM)	Length of induction (days)
M1	0.1	0	21
M2	0.1	10	28
M3	0.1	60	35
M4	5	60	28
M5	5	0	35
M6	5	10	21
M7	10	10	35
M8	10	60	21
M9	10	0	28

M1—control conditions, M2–M9—variations for testing the Taguchi method

#### Experiment 2

In vitro plant regeneration via anther culture used the same protocol as for preparing donor plants. In Experiment 2 three trials were designed: control trial (M10) and trials which utilised the optimised concentrations of CuSO_4_, AgNO_3_ and length of induction conditions to generate the lowest percentage of differences (based on TTCIV) between donor and regenerant plants (M12), and the highest percentage of differences between donor and regenerant plants (M13) (Table 2).

**Table 2:** Optimised induction trials for in vitro plant regeneration of barley via androgenesis (anther cultures)

Trial	Optimised factors		
	CuSO_4_ (µM)	AgNO_3_ (µM)	Length of induction (days)
M10	0.1	0	21
M12	10	60	21
M13	2.95	15	28

M10—control, M12 and M13—the lowest and highest percentage of differences, respectively, between donor and regenerant plants based on total tissue culture-induced variation (TTCIV)

### Molecular analysis

#### Genomic DNA extraction

Total genomic DNA was extracted from 100 mg fresh leaves of donor and regenerant plants obtained via androgenesis (Experiments 1 and 2) using the DNeasy MiniPrep kit (Qiagen). DNA quantity was evaluated spectrophotometrically, and DNA integrity and purity were verified electrophoretically.

#### MetAFLP genotyping

The metAFLP protocol was described elsewhere (Bednarek et al. 2007) with slight modifications (Machczyńska et al. 2014b). DNA samples were digested with Acc65I/MseI and KpnI/MseI endonucleases following adaptor ligation and pre-selective and selective amplification steps (Online Resource Table S1). After the ligation step, the reaction mixtures were diluted with water (1:3, v/v). The pre-selective PCR product before selective amplification also was diluted with water (1:20, v/v). Selective amplification was conducted in the presence of ^32^P-labelled selective primers. After fractioning the selective PCR product with 7% polyacrylamide gel electrophoresis (PAGE), DNA fragments were visualised by exposing to X-ray film.

#### Evaluating markers related to DNA methylation pattern and sequence changes

The molecular profiles resulting from the Acc65I/MseI (A) and KpnI/MseI (K) metAFLP platforms were juxtaposed and converted into a ‘0–1’ binary matrix. The Acc65I/MseI platform can identify DNA sequence and methylation changes, whereas the KpnI/MseI platform exclusively identifies sequence changes. Thus, markers correlated with DNA methylation status can be identified (M). Namely, markers that were present in the first (A) and missed in the second (K) metAFLP platform (or vice versa) were related to DNA methylation (M) (Chwedorzewska and Bednarek 2012). Thus, data from the Acc65I/MseI-KpnI/MseI (M) metAFLP platform were used to evaluate methylation markers, whereas data from the KpnI/MseI platform were used to evaluate DNA sequence variation.

#### MetAFLP quantitative characteristics

The juxtaposed DNA profiles generated after PAGE separation of the PCR products amplified from DNA samples digested with Acc65I/MseI and KpnI/MseI platforms were used to assess quantitative metAFLP characteristics (Bednarek et al. 2007; Machczyńska et al. 2014b). The following parameters were evaluated: TTCIV, sequence variation (SV), demethylation (DMV), de novo methylation (DNMV) and changes in methylation (dMET, calculated as the difference between de novo methylation and demethylation).

#### Quantifying symmetric and asymmetric methylation change based on metAFLP analysis

The Acc65I–KpnI restriction sequence is 5′-GGATCC-3′; thus selective primers ending with any combination of A and T would amplify the CpXpX asymmetric sequence. Primers ending with the XG sequence at the 3′-ends of selective metAFLP primers and those with the CG sequence reflect symmetric sequences (the list of respective primers is presented in Online Resource Table S2). Thus, ‘events’ reflecting methylation changes can be identified using markers amplified by selective primers for symmetric and asymmetric sequences (Bednarek et al. 2007; Machczyńska et al. 2014b, 2015). All formulas used for the quantification of ‘events’ within sequence contexts are as described earlier (Bednarek et al. 2007; Machczyńska et al. 2014b; Machczyńska et al. 2015). The only difference between this study and the earlier studies is that the expression in the denominator 1 (D1(‘0000’)) is identical in all cases and assumes a total number of ‘events’ (including ‘0000’) independently of the symmetric and asymmetric sequence context. The final quantitative characteristics of each sequence context were weighted by the number of markers evaluated for the context and marked in the end of _CN (Table 3). The excel file used for calculations can be assessed on Online Resource ESM_1 and ESM_2).

**Table 3:** Formulae for quantification of demethylation variation (DMV), de novo methylation variation (DNMV) and sequence variation (SV) within the CXX, CG and CXG methylation context

Code	Formula
Z_E	Z_0000 + Z_0001 + Z_0010 + Z_0011 + Z_0100 + Z_0101 + Z_0110 + Z_0111 + Z_1000 + Z_1001 + Z_1010 + Z_1011 + Z_1100 + Z_1101 + Z_1110 + Z_1111
Z_SE	Z_0001 + Z_0010 + Z_0101 + Z_0110 + Z_1001 + Z_1010 + Z_1101 + Z_1110
Z_DME	Z_0110 + Z_0111
Z_DNME	Z_1001 + Z_1011
Z_CE	Z_0100 + Z_1000
Z_SNMSs	Z_1100 + Z_1101 + Z_1111
Z_SMSs	Z_0011 + Z_1101
Z_TTCIE	Z_SE + Z_DME + Z_DNME + Z_CE
CXX_D1 ('0000')	CXX_SE + CXX_DME + CXX_DNME + CXX_CE + CXX_SNMSs + CXX_SMSs + CXX_'0000'
CG_D1 ('0000')	CG_SE + CG_DME + CG_DNME + CG_CE + CG_SNMSs + CG_SMSs + CG_'0000'
CXG_D1 ('0000')	CXG_SE + CXG_DME + CXG_DNME + CXG_CE + CXG_SNMSs + CXG_SMSs + CXG_'0000'
D1 ('0000')	CXX_D1 ('0000') + CG_D1 ('0000') + CXG_D1 ('0000')
Z_DMV	100 × Z_DME/D1 ('0000')
Z_DNMV	100 × Z_DNME/D1 ('0000')
Z_SV	100 × Z_SE/D1 ('0000')
Z_CV	100 × Z_CE/D1 ('0000')
Z_TTCIV	100 × Z_TTCIE/D1 ('0000')
Z_SV_CN	Z_SV + [Z_SV × Z_CV/(Z_SV + Z_DMV + Z_DNMV)]/Z_E
Z_DMV_CN	Z_DMV + [Z_DMV × Z_CV/(Z_SV + Z_DMV + Z_DNMV)]/Z_E
Z_DNMV_CN	Z_DNMV + [Z_DNMV × Z_CV/(Z_SV + Z_DMV + Z_DNMV)]/Z_E
Z_dMET_CN	Z_DNMV_CN-Z_DMV_CN
Methylation context	3′-ends of selective primers
CXX	AAA, AAT, ATA, ATT, TAA, TAT, TTA, etc
CG	CGG, CGA, CGT, CGC, GCG, GCA, GCT, GCC, etc.
CXG	AAG, AAC, ATG, ATC, TAG, TAC, TTG, etc.

D1 (‘0000’) states for the denominator 1; _C—correction for complex variation, Z—methylation context or without context depending on the marker set used for quantification, E—number of ‘events’

### Statistics

#### Characterising metAFLP markers

GenAlEx6.501 (Excel add-in software) was used to estimate the percentage of polymorphic loci (*%P*) generated by metAFLP platforms for donor plants (Peakall and Smouse 2012). Shannon’s Information Index (*I*) was used to characterise marker system informativeness.

#### Agglomeration analysis

Agglomeration analysis based on metAFLP markers was conducted in PAST software (Schönswetter and Tribsch 2005). UPGMA and Jaccard index of similarity measure with 999 bootstrap replicates were used to estimate the robustness of the branches.

#### Analysis of molecular variance (AMOVA)

The metAFLP markers evaluated using the KpnI/MseI platform and those addressed to methylation changes (Acc65I/MseI–KpnI/MseI) were subjected to AMOVA using the GenAlEx6.501 Excel add-in to generate the *Φ*_*PT*_ index.

#### Testing for outliers

Regenerated plants from Experiment 1 and Experiment 2 were evaluated for the presence of outliers using the Z_TTCIV (Z represents all contexts taken together) metAFLP quantitative characteristic and XLSTAT 2018.1.49205 software. Grubbs’ test was conducted on metAFLP characteristics at significance level of 5%.

#### Taguchi method

The metAFLP TTCIV characteristics evaluated for each of the M2–M9 trials (Experiment 1) were imported into the QI Macros230T (Excel add-in software, KnowWare International, Inc.) to identify optimised conditions for plant regeneration with minimum and maximum percentages of sequence and DNA methylation changes compared with the donor plants.

#### Analysis of variance (ANOVA)

Differences between M1–M9 (Experiment 1) and M10–M13 (Experiment 2) trials were determined via ANOVA followed by Tukey’s honestly significant difference test using different metAFLP characteristics (Z_TTCIV; Z_SV_CN; Z_DMV_CN; Z_DNMV_CN; Z_dMET_CN; Z states for CXX, CG, CXG, context-derived markers or all markers) separately or in combination. The differences were significant at *p* ≤ 0.05 and α = 0.01. The calculations were performed using XLSTAT 2018.1.49205 Excel add-in software.

#### Pearson correlation

Pearson correlation analysis between Z_SV_CN and Z_dMET_CN/Z_DMV_CN/Z_DNMV_CN (Z represents all contexts taken together) was performed using XLSTAT 2018.1.49205 software and data from Experiment 2.

## Results

### Donor plants characteristics and explant tissue

Thirty-six morphologically uniform donor plants (Ds), which were the progeny of double haploid plants derived via anther cultures, were prepared as a source of explants for Experiments 1 and 2. MetAFLP profiling of Ds with 13 selective primer combinations (Online Resources Table S2) identified 348 markers that were shared between the Acc65I/MseI–KpnI/MseI (M) and KpnI/MseI (K) data. The first platform (M) amplified as many as 20 AFLPs, whereas the second platform (K) amplified seven polymorphic AFLPs. The percentages of polymorphic loci (*%P*) in M and K platforms were 5.75% and 2.01%, respectively. Shannon's information index (*I*) in M and K platforms amounted to 0.023 and 0.006, respectively. Finally, from the prepared pool of donor plants, one plant was chosen for Experiment 1 (optimisation), and six plants were chosen for Experiment 2 (verification).

### Experiment 1: optimisation

#### Molecular profiling and marker system characteristics

A total of 43 double haploid (DH) regenerants were derived via anther cultures in Experiment 1. The regenerants were morphologically similar to the donor plant and were fully fertile.

Amplification of the DNAs extracted from DH regenerants and their respective donor plant with 13 selective primer combinations (Online Resources Table S2) resulted in 407 AFLPs shared among the M1–M9 tissue culture tests. The percentage of polymorphic loci (*%P*) varied among these trials from 0 to 6.39 for the Acc65I/MseI-KpnI/MseI (M) AFLPs and from 0.49 to 3.69 for the KpnI/MseI (K) AFLPs. Shannon’s Information Index (*I*) for the M and K AFLPs ranged from 0 to 0.032 and 0.002 to 0.017, respectively. The donor plant profiles were omitted in the calculation of *%P* and *I*. The values of all polymorphic and Shannon’s information indices are given in Online Resource Table S3.

#### Agglomeration analysis

**Fig. 1:**
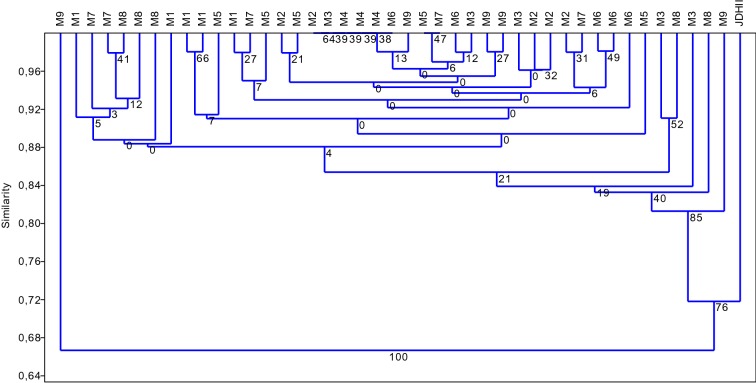
Agglomeration analysis (UPGMA) based on Jaccard similarity coefficients of Acc65I/MseI–KpnI/MseI (M) AFLPs identifies site DNA methylation changes in anther-derived regenerants of Experiment 1. Regenerants from the M1–M9 trials and the donor plant (JDHII) are included. Bootstrap values are indicated at the nodes

Cluster analysis of the anther-derived regenerants based on Jaccard similarity coefficients of Acc65I/MseI-KpnI/MseI (M) AFLPs demonstrates that essentially all regenerants fall into the same group (based on bootstrap value). Only one regenerant and the donor plant were outside of the cluster. The regenerants were 84% similar to each other (Fig. 1).

**Fig. 2:**
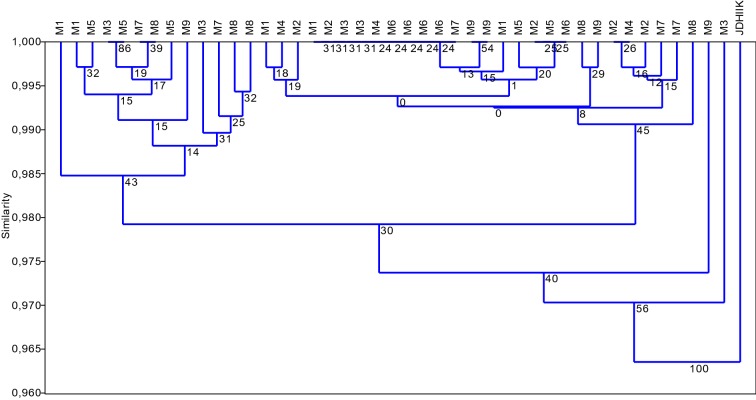
Agglomeration analysis (UPGMA) based on Jaccard similarity coefficients of KpnI/MseI (K) AFLPs identifies DNA sequence changes in anther-derived regenerants of Experiment 1. Regenerants derived in the M1–M9 trials and the donor plant (JDHIIK) are included. Bootstrap values are indicated at the nodes

The grouping derived from KpnI/MseI (K) AFLP markers is consistent with that derived from Acc65I/MseI-KpnI/MseI (M) markers; however, the differences among K-platform regenerants were lower than those in M-platform regenerants. Only one regenerant and the donor plant were outside of the cluster. The regenerants were 98% similar to each other (Fig. 2).

#### Analysis of molecular variance

AMOVA was performed on the metAFLPs amplified from DNA of fertile regenerants derived via anther cultures in Experiment 1 (M1–M9). The results showed that up to 15.7% of the explained molecular variance was due to difference among trials of the Acc65I/MseI-KpnI/MseI (M) AFLP markers (Φ_*PT*_ value = 0.157, *p* = 0.001). Similar analyses of the KpnI/MseI (K) AFLP markers accounted for 10.6% of the explained variance (Φ_*PT*_ value = 0.106, *p* = 0.034).

#### MetAFLP characteristics and identifying outlier plants in Experiment 1

The metAFLP markers enabled evaluation of numerous ‘events’ (Online Resources Table S4) and identified the variation types within the M1–M9 trials (Experiment 1). The most common ‘events’ were assigned to the ‘Z_1111’ pattern. All ‘event’ types were observed in Experiment 1, but they were not observed in individual trials.

The analysis of outlier plants was performed using the Z_TTCIV (Z represents all contexts taken together) formula characteristics. The results indicated that all except a single regenerant from the M4 trial fit within the given set (Online Resource Table S5) because the Grubbs test *p* value was > 0.05; only one regenerant had *p *< 0.05. Thus, this one M4 outlier was excluded from further analysis.

#### Analysis of variance based on metAFLP characteristics

The differences among M1–M9 trials were evaluated based on metAFLP characteristics. All of them except Z_DNMV_CN, were significant (Table 4). MetAFLP characteristics were evaluated based on ‘events’ (site DNA methylation and sequence changes affecting Acc65I-KpnI restriction sites) showing that the Z_TTCIV and Z_SV_CN formulae were predominant in the M8 trial (Table 4), and were two- to three-times higher than those in other trials. The lowest Z_DMV_CN value was observed in M8, whereas the highest Z_DMV_CN value was observed in the M5, M7 and M9 trials. The Z_DNMV_CN values for all trials were essentially identical and ranged from 0.69–0.79. The Z_dMET_CN values were essentially all negative; the only positive Z_dMET_CN value was in the M8 trial.

Tukey’s test of Z_TTCIV showed that the M8 trial was distinct from all other trials that formed a joint group (Table 4). The Z_TTCIV value of the M8 trial was two- to three-times higher than in the other trials. The similar grouping was identified by the Z_SV_CN, Z_DMV_CN and Z_dMET_CN characteristics.

**Table 4:** ANOVA statistics for each of the metAFLP characteristics and arrangement of the average metAFLP characteristics evaluated for the M1–M9 trials. Z represents all sequence contexts taken together

		metAFLP characteristic				
		Z_TTCIV	Z_SV_CN	Z_DMV_CN	Z_DNMV_CN	Z_dMET_CN
ANOVA	F	49.434	119.666	24.368	0.773	23.380
	*p*	0.0001	0.0001	0.0001	0.629	0.0001
Trials	M1	6.53^b^	2.96^b^	1.48^a^	0.74^a^	− 0.74^b^
	M2	6.20^b^	2.36^b^	1,48^a^	0.74^a^	− 0.74 ^b^
	M3	7.03^b^	3.42^b^	1.48^a^	0.69^a^	− 0.79^b^
	M4	5.86^b^	2.32^b^	1.39^a^	0.70^a^	− 0.70^b^
	M5	6.85^b^	3.15^b^	1.53^a^	0.74^a^	− 0.79^b^
	M6	5.74^b^	2.50^b^	1.11^a^	0.70^a^	− 0.42^b^
	M7	6.62^b^	3.15^b^	1.53^a^	0.74^a^	− 0.79^b^
	M8	15.43^a^	13.03^a^	0.00^b^	0.79^a^	0.79^a^
	M9	7.32^b^	3.20^b^	1.53^a^	0.70^a^	− 0.84^b^

M1—control, M2–M9—optimisation trials. The Z_TTCIV, Z_SV_CN, Z_DMV_CN, Z_DNMV_CN and Z_dMET_CN characteristics reflect total tissue culture-induced variation, sequence variation, demethylation, de novo methylation and change in DNA methylation, respectively. The a, b and c superscript letters indicate Tukey’s grouping. The presented data do not include outliers that were excluded based on Grubs test

### Experiment 2: verifying conditions suggested by the Taguchi method

#### Molecular profiling and marker system characteristics

The in vitro anther culture plant regeneration trials (M12 and M13) with optimised concentrations of CuSO_4_, AgNO_3_ and tissue culture induction period (Table 2) resulted in 19 regenerants that were morphologically identical to donor plants and those in the M10 trial. The M10, M12 and M13 trials resulted in 4, 7 and 8 regenerants, respectively.

The metAFLP genotyping of regenerants and donors identified 306 markers that were shared between the Acc65I/MseI–KpnI/MseI (M) and KpnI/MseI (K) platforms for the M10, M12 and M13 trials. The respective percentage of polymorphic loci (*%P*) and Shannon’s information indices (*I*) are listed in Online Resources Table S6. The *%P* value was highest in the M13 trial, reflecting optimisation towards maximum differences between donor and regenerant plants. The *%P* value was lowest in the M10 trial, reflecting control conditions for the Acc65I/MseI-KpnI/MseI (M) platform. For the KpnI/MseI (K) AFLP markers, the *%P* value was highest in the M13 trial and lowest in the M12 trial, which was designed to minimise differences between donor and regenerant plants. Shannon’s *I* values ranged from 0.02 (M12) for KpnI/MseI (K) markers to 0.062 (M13) for Acc65I/MseI–KpnI/MseI (M) markers (Online Resource Table S6).

#### Agglomeration analysis

**Fig. 3:**
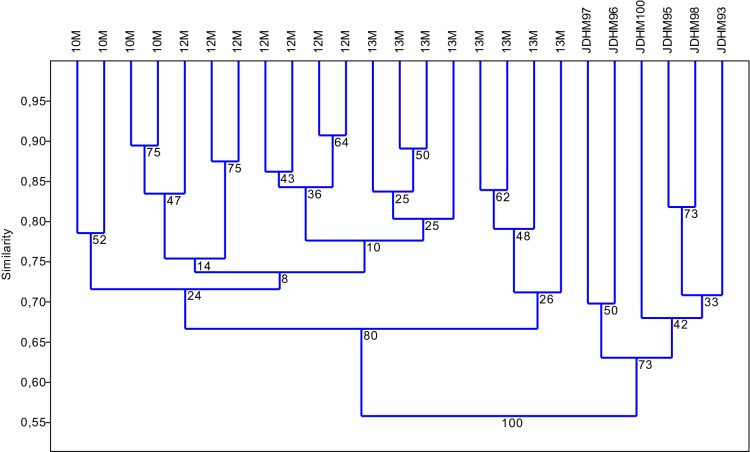
Agglomeration analysis (UPGMA, Jaccard) of metAFLPs related to DNA methylation change (Acc65I/MseI–KpnI/MseI, M) for the M10–M13 trials. The M10 trial mirrors control conditions. The M12 and M13 regenerants were derived according to the optimised conditions for minimum and maximum differences between donor and regenerant plants, respectively. Bootstrap value is indicated on the nodes

Agglomeration analysis was conducted on the metAFLPs related to DNA methylation change (M) (Acc65I/MseI-KpnI/MseI) in the M10–M13 trials (Fig. 3). The results indicated that, according to the bootstrap value, all donors (source tissues) for the in vitro tissue culture-regenerated plants formed a single cluster, whereas the regenerants formed a second cluster with two subclusters. The first subcluster encompasses the M10 and M12 regenerants and some M13 regenerants. The second subcluster encompasses the M13 regenerants. Donors exhibit the highest levels of variation.

**Fig. 4:**
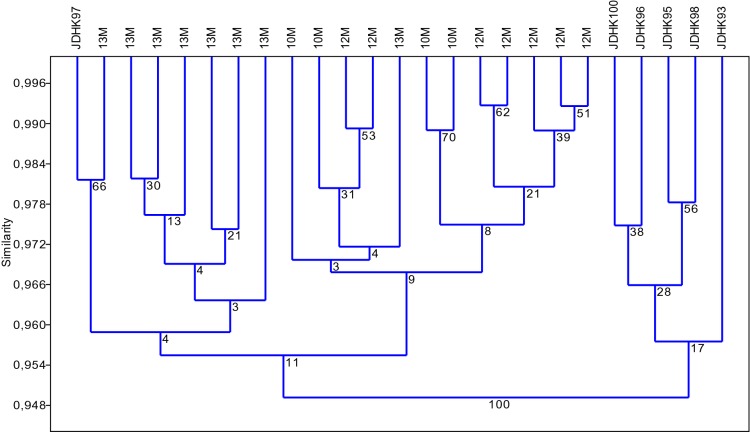
Agglomeration analysis (UPGMA, Jaccard) of metAFLPs related to the DNA mutations (KpnI/MseI) (K) for the M10–M13 trials. The M10 trial mirrors control conditions. The M12 and M13 regenerants were derived according to the optimised conditions for minimum and maximum differences between donor and regenerant plants, respectively. Bootstrap value is indicated on the nodes

Cluster analysis performed on the KpnI/MseI (K) markers (Fig. 4) also resulted in two groups: the first one encompassed all regenerants and one donor plant, whereas the second encompassed the remaining Ds. Higher variation levels were observed in the first cluster and reflected differences among regenerants. The second cluster grouped into two subclusters: one subcluster included essentially all M13 regenerants and one donor plant; one subcluster aggregated the M10 and M12 regenerant plants. The variation exhibited by the KpnI/MseI (K) markers was generally lower than that of the Acc65I/MseI-KpnI/MseI (M) markers.

#### Analysis of molecular variance

AMOVA demonstrated that up to 20.6% of observed variation was due to differences among trials related to methylation changes in Acc65I/MseI–KpnI/MseI (M) AFLPs (Φ_*PT*_ value = 0.206, *p* = 0.001) used for the verification process in Experiment 2. Similar data for DNA sequence changes using the KpnI/MseI (K) AFLPs equalled 25.6% (Φ_*PT*_ value = 0.256, *p* = 0.001).

#### MetAFLP characteristics and identifying outliers in Experiment 2

Conversion of the metAFLP markers obtained for donor and regenerant plants in the M10–M13 trials showed that Z_‘1111’ was the most frequent, whereas Z_‘0010’, reflecting DNA sequence changes, is the rarest in all trials (Online Resource Table S7).

The metAFLP Z_TTCIV (Z represents all sequence contexts together) characteristic generated in Experiment 2 was subjected to the Grubbs test but failed to identify any outliers (data not shown).

#### Analysis of variance based on metAFLP characteristics

In general, the highest values of Z_TTCIV, Z_SV_CN, Z_DMV_CN and Z_DNMV_CN were obtained in the M10 trial (control), whereas the lowest values were obtained in the M12 trial designed for minimum differences between donor and regenerant plants. For the M13 trial, all metAFLP characteristics were slightly lower than the values in the M10 trial (Table 5).

**Table 5:** ANOVA statistics for each of the metAFLP characteristics in the verification process and averaged metAFLP characteristics evaluated for the M10, M12 and M13 trials

		metAFLP characteristic				
		Z_TTCIV	Z_SV_CN	Z_DMV_CN	Z_DNMV_CN	Z_dMET_CN
ANOVA	F	2.400	0.759	1.009	0.639	0.671
	*p*	0.123	0.484	0.387	0.541	0.525
Trial	M10	11.37^a^	4.30^a^	1.77^a^	2.92^a^	0.69^a^
	M12	9.69^a^	3.84^a^	1.50^a^	2.45^a^	0.40^a^
	M13	10.31^a^	4.06^a^	1.24^a^	2.78^a^	0.61^a^

M10—control; M12 and M13—verification trials. Z_TTCIV, Z_SV_CN, Z_DMV_CN, Z_DNMV_CN and Z_dMET_CN reflect total tissue culture-induced variation, sequence variation, demethylation, de novo methylation and change in DNA methylation, respectively. The a, b and c superscript letters indicate Tukey’s grouping. Z represents all sequence contexts taken together

Despite quantitative differences among the M10–M13 trial evaluations based on metAFLP characteristics (Z_TTCIV, Z_SV_CN, Z_DNMV_CN, Z_DMV_CN and Z_dMET_CN), models that assumed distinctiveness of the M10–M13 trials for any characteristics were not significant. Tukey’s test failed to discriminate between the M10–M13 trials (Table 5).

#### Methylation context

The metAFLP genotyping distinguished symmetric (CG and CXG) and asymmetric (CXX) methylation contexts. Comparison of the methylation contexts in Z_DMV_CN and Z_DNMV_CN revealed significant differences among contexts (without assuming that the trials were significant), and showed that CG was affected by most methylation changes (Table 6). Tukey’s test indicated that CXG and CXX formed a single group, whereas CG formed the second group in Z_DMV_CN. All methylation contexts considered for de novo methylation differed. Differences between contexts also were observed for Z_dMET_CN, with CXX forming a separate group.

Comparison of Z_DMV_CN and Z_DNMV_CN within symmetric and asymmetric methylation contexts showed that Z_DNMV_CN prevailed over Z_DMV_CN for CG and CXG. Within CG and CXG contexts, Z_DNM_CN and Z_DNMV_CN differed as indicated by ANOVA and confirmed by Tukey’s test. ANOVA demonstrated no significant differences between Z_DMV_CN and Z_DNMV_CN due to the CXX methylation context (Table 6).

**Table 6:** ANOVA and Tukey’s grouping of methylation contexts within DMV and DNMV as well as grouping of differences between Z_DMV_CN and Z_DMV_CN within CG, CXG and CXX methylation contexts when trials were not distinguished

		metAFLP characteristic (%)				Methylation context				
		Z_DMV_CN	Z_DNMV_CN	Z_dMET_CN				CG	CXG	CXX
ANOVA	F	71.239	108.7470	6.615	ANOVA		F	17.789	28.512	0.004
	*p*	0.0001	0.0001	0.003			*p*	0.000	0.0001	0.951
Methylation context	CG	1.19^a^	1.88^a^	0.68^a^	metAFLP characteristic(%)		Z_DMV_CN	1.19^b^	0.18^b^	0.08^a^
	CXG	0.18^b^	0.73^b^	0.55^a^						
	CXX	0.08^b^	0.08^c^	0.00^b^			Z_DNMV_CN	1.88^a^	0.73^a^	0.08^a^

Superscript letters indicate Tukey’s grouping.

ANOVA indicated that the differences among M10–M13 trials for each methylation context and within methylation pattern changes (Z_DMV_CN and Z_DNMV_CN) were significant for CXX in the case of Z_DMV_CN, Z_DNMV_CN and Z_dMET_CN, which was confirmed by Tukey’s test (Table 7). Despite the lack of Tukey’s grouping, the metAFLP characteristics in the M12 trial, such as Z_DMV_CN, Z_DNMV_CN and Z_dMET_CN within methylation contexts, were lower than in the control (except for Z_DNMV_CN and Z_dMET_CN in the CG context). The differences between M10 and M12 were due to Z_DNMV_CN and Z_dMET_CN in the CXX context. Significant differences between M10 and M13 were assigned to the CXX context for Z_DMV_CN and Z_DNMV_CN, and to Z_DMV_CN for CXG. A similar analysis for the M12 and M13 trials showed that differences also were related to Z_DMV_CN in CG and CXX, and to Z_DNMV_CN in the CXX methylation context. No differences were observed between the trials for the CXG methylation context.

**Table 7:** ANOVA and Tukey’s grouping of the M10–M13 trials by methylation context in Z_DNMV_CN and Z_DMV_CN

Methylation context		CG			CXG			CXX		
metAFLP characteristic		Z_DMV_CN	Z_DNMV_CN	Z_dMET_CN	Z_DMV_CN	Z_DNMV_CN	Z_dMET_CN	Z_DMV_CN	Z_DNMV_CN	Z_dMET_CN
ANOVA	F	2.274	0.401	1.146	0.930	1.637	0.671	7.719	5.057	4.023
	p	0.135	0.677	0.343	0.415	0.225	0.525	0.005	0.020	0.038
Trial	M10	1.47a	1.69a	0.22a	0.31a	1.00a	0.69a	0.00b	0.23a	0.23a
	M12	1.33a	1.87a	0.54a	0.17a	0.58a	0.40a	0.00b	0.00b	0.00b
	M13	0.94a	1.98a	1.04a	0.11a	0.72a	0.61a	0.19a	0.08ab	− 0.11ab
ANOVA	F	0.201	0.288	0.281	0.580	5.259	1.194	–	17.168	17.168
	p	0.664	0.604	0.609	0.466	0.048	0.503	–	0.003	0.003
Trial	M10	1.47a	1.69a	0.22a	0.31a	1.00a	0.69a	–	0.23a	0.23a
	M12	1.33a	1.87a	0.54a	0.17a	0.58b	0.40a	–	0.00b	0.00b
ANOVA	F	2.463	0.792	1.621	6.055	1.087	0.096	5.555	2.921	5.027
	p	0.148	0.394	0.232	0.034	0.322	0.763	0.040	0.118	0.049
Trial	M10	1.47a	1.69a	0.22a	0.31a	1.00a	0.69a	0.00b	0.23a	0.23a
	M13	0.94a	1.98a	1.04a	0.11b	0.72a	0.61a	0.19a	0.08a	-0.11b
ANOVA	F	5.867	0.162	1.431	0.210	0.569	0.778	10.110	2.022	1.063
	p	0.031	0.694	0.253	0.655	0.464	0.394	0.007	0.179	0.321
Trial	M12	1.33a	1.87a	0.54a	0.17a	0.58a	0.40a	0.00b	0.00a	0.00a
	M13	0.94b	1.98a	1.04a	0.11a	0.72a	0.61a	0.19a	0.08b	− 0.11a

Superscript letters reflect Tukey’s grouping.

#### Pearson correlation

The Pearson correlation between Z_SV_CN and Z_dMET_CN (for all trials taken together) evaluated in Experiment 2 without analysing methylation contexts (CG, CXG, CXX) was positive (*r* = 0.033), whereas the coefficient of determination equalled 0.001 but was insignificant (*p* = 0.894). When methylation contexts but not trials were considered, no correlation between Z_SV_CN and Z_dMET_CN, Z_DMV_CN or Z_DNMV_CN was found (Table 8). Analysing this correlation within methylation contexts detected correlation between Z_SV_CN and Z_dMET_CN, Z_DMV_CN and Z_DNMV_CN depending on the trial. In the CG context in the control trial (M10), positive correlation of Z_SV_CN and Z_DMV_CN and negative correlation between Z_SV_CN and Z_dMET_CN were evaluated; Z_SV_CN–Z_DMV_CN and Z_SV_CN–Z_dMET_CN correlations were significant at *p* = 0.1. However, these correlations changed to insignificant for the M13 trial (optimised for maximum variation). The only significant correlation in the CXG context was detected in the case of Z_SV_CN and Z_DNMV_CN for the M13 trial. Negative Z_SV_CN–Z_dMET_CN and Z_SV_CN–Z_DNMV_CN correlations were detected in the M10 and M13 trials in the CXX context. A positive Z_SV_CN–Z_DMV_CN correlation was detected in the M13 trial for the CXX context. No correlations of Z_SV_CN and Z_dMET_CN, Z_DMV_CN or Z_DNMV_CN were detected for the M12 trial (optimised for minimum variation) (Table 8).

**Table 8:** Pearson correlation for sequence changes (Z_SV_CN) and difference in methylation (Z_dMET_CN), demethylation (Z_DMV_CN), and de novo methylation (Z_CN) in symmetric and asymmetric methylation contexts in all trials and in individual trials

Trials	Methylation context	CG			CXG			CXX		
	metAFLP characteristics	Z_SV_CN-Z_dMET_CN	Z_SV_CN-Z_DMV_CN	Z_SV_CN-Z_DNMV_CN	Z_SV_CN-Z_dMET_CN	Z_SV_CN-Z_DMV_CN	Z_SV_CN-Z_DNMV_CN	Z_SV_CN-Z_dMET_CN	Z_SV_CN-Z_DMV_CN	Z_SV_CN-Z_DNMV_CN
All trials	Correlation matrix	0.000	0.017	0.017	0.269	0.037	0.323	− 0.205	0.022	0.311
	*p*-values	0.999	0.944	0.945	0.265	0.881	0.177	0.399	0.929	0.195
M10	Correlation matrix	− 0.963	0.989	− 0.900	0.530	− 0.099	0.526	− 0.956	–	− 0.956
	*p*-values	0.037	0.011	0.100	0.470	0.901	0.474	0.044	0	0.044
M12	Correlation matrix	− 0.066	0.157	− 0.025	0.285	− 0.142	0.330	–	–	–
	*p*-values	0.887	0.736	0.958	0.536	0.761	0.470	0	0	0
M13	Correlation matrix	0.625	− 0.694	0.553	0.522	0.583	0.728	–0.818	0.714	− 0.818
	*p*-values	0.098	0.056	0.156	0.184	0.129	0.041	0.013	0.047	0.013

## Discussion

Tissue culture is an essential technique of plant propagation and development, and has been used to generate doubled haploids (Morrison and Evans 1988) and transformed plants (Husaini et al. 2010), for conservation (Li and Pritchard 2009) and to produce new varieties (Patial et al. 2019). Protocols have been developed for the regeneration of crop and model plant species that speed up the time of obtaining plants (Patial et al. 2019), eliminate disease (García-Gonzáles et al. 2010) and continually produce hundreds of thousands of regenerants. Although plant regeneration via tissue culture has many advantages, it triggers tissue culture-induced variation that is shared among regenerants (Bednarek et al. 2007; Fiuk et al. 2010; Machczyńska et al. 2015) and subsequently transmitted to the progeny (Han et al. 2018). The level of induced variation may significantly impact the homogeneity of regenerants (Coronel et al. 2018), which may adversely affect evaluated materials. By contrast, increasing variation via tissue culture can be beneficial, as tissue cultures enable the introduction of new variants without utilising genetically modified organisms (Bittsánszky et al. 2009). Thus, it is crucial to determine how tissue cultures components (or other factors) can be modified to regulate the level of variability according to specific applications.

The goal of this study was to verify whether tissue culture-induced variation can be regulated by modifying media ingredients and time span for plant regeneration. First, the uniformity of donor plants used for the optimisation and verification experiments needed to be ensured. This could be achieved using plant materials derived via androgenesis, as any double haploid originates via doubling haploid genome (Ohnoutkova et al. 2019; Sarao and Gosal 2018). However, tissue cultures can induce different variation levels depending on the genotype (Yu et al. 2011), explant source (Krishna et al. 2016), regeneration method (Dey et al. 2015; Saravanan et al. 2011; Smulders and de Klerk 2011) and media ingredients (Rodríguez López et al. 2004; Shooshtari et al. 2018). The so-called pre-existing variation also may affect shared variation levels among regenerants (Evans et al. 1984; Karp 1994). It is assumed that variation induced in regenerants may be partly inherited by generative progeny (Han et al. 2018). Thus, regenerants subjected to generative cycle(s) should produce more uniform plants than regenerants (Machczyńska et al. 2014b, 2015; Orłowska et al. 2016, 2020). Our recent studies demonstrated that, depending on the species and the number of generative cycles, the genome stabilisation level may vary (Machczyńska et al. 2014a). Thus, to deliver donor plants with an acceptable level of uniformity, our donor plants originated from anther-derived DH regenerants that underwent a single self-pollination step. The approach allowed us to obtain a set of donor plants with as little as 5.75% and 2.01% of polymorphic metAFLP loci related to methylation and sequence changes, respectively. To avoid differences among regenerants, only a single donor plant was used as the source of explants for further experiments.

The optimisation experiment assumes that three factors affecting tissue culture-induced variation are involved simultaneously. To minimise the number of samples required for the experiment, we used Taguchi’s method (Taguchi 1986), which allows the experimental design to be reduced to eight trials. In each case, different concentrations of AgNO_3_ and CuSO_4_ and different time spans in induction tissue culture medium are tested at three levels to regenerate plants via anther cultures. This strategy has been used in biotechnological experiments (Yari and Mostafaie 2011) and recently to optimise the number of green plants derived via androgenesis in cereals (Orłowska et al. 2020). In the given design, we were interested to maximise or minimise the level of differences in DNA sequences and site DNA methylation patterns between donor and regenerant plants. The differences could be measured using a metAFLP method and quantitative characteristics including TTCIV and its counterparts (de novo methylation, demethylation and sequence variation) (Machczyńska et al. 2014b). Based on metAFLP markers related to DNA methylation pattern, the differences revealed among regenerants ranged from 0 to 6.39%, whereas DNA sequence differences ranged from 0.25 to 3.69% of polymorphic loci depending on optimisation design. This level of variability is higher than that of the donor plants. Our results demonstrated that varying the three factors enabled us to obtain regenerants that differed from the donor plant.

Cluster analysis showed that regenerants derived from M1–M9 trials were mostly mixed for methylation-related and sequence-related metAFLP markers. The differences among trials were higher in the case of methylation (15.7%) than sequence (10.6%) markers, as indicated by AMOVA. The presented data support the notion that both DNA methylation alterations and sequence changes are induced during tissue culture-mediated plant regeneration (Coronel et al. 2018; Gimenez et al. 2016), and they may result from varying in in vitro tissue culture conditions. TTCIV evaluated from specific metAFLP ‘events’ did not identify outliers (i.e., regenerants that did not fit in the trial), so all regenerants were used for further experiments.

The metAFLP Z_TTCIV characteristics evaluated for the M2–M9 trials in combination with Taguchi’s method indicated that trial conditions might have produced anther culture regenerants with maximum and minimum number of differences in regenerants compared with the donor plant. For the trial designed to produce a minimum number of differences between donor and regenerant plants (M12), the CuSO_4_ and AgNO_3_ concentrations changed compared with control conditions, whereas the time in tissue culture was not a significant factor. For the trial designed to produce a maximum number of differences between donor and regenerant plants (M13), all factors changed compared with control conditions. Assuming that time in tissue culture is the responsible factor that induces variation (longer culture time leads to higher variation) (Noro et al. 2007), higher variation levels were expected among M13 regenerants. By contrast, the roles of CuSO_4_ and AgNO_3_ in tissue culture-induced variation were not evident. Plant regeneration in the M12 trial occurs under the highest CuSO_4_ (10 µM) and AgNO_3_ (60 µM) concentrations used in this experiment. Previous studies indicated that this CuSO_4_ concentration in barley tissue culture improved the anther response by 15% and the number of green androgenetic plants by 400% (Wojnarowiez et al. 2002), and increased the number of regenerated plantlets (Jacquard et al. 2009). Elevation of Cu^2+^ ions to 40 µM dramatically reduced the number of responding anthers (Jacquard et al. 2009). There are no reports on the effect of 10 µM Cu on tissue culture-induced variation, but 80 µM CuSO_4_ can cause harmful effects in *Abies nordmanniana* cells by generating a significant excess of ROS (Nawrot-Chorabik 2017). Supplementing culture media with AgNO_3_ promoted embryogenesis and prevented callus necrosis in wheat (Wu et al. 2006). However, 60 µM AgNO_3_ significantly suppressed embryogenic calli formation due to silver toxicity to immature wheat embryos cultured in vitro (Wu et al. 2006). We used 60 µM AgNO_3_ along with CuSO_4_, which reduced tissue culture-induced variation compared with control (M10), but still generated significant variability. The levels of factors for optimising media for maximum differences between regenerant and donor plants (M13) used a lower salt concentration than those used in the control (M10) trial. The significant factor affecting variability was the time in anther culture induction media (28 days in M13 vs. 21 days in M10 and M12).

The numbers of polymorphic loci related to site DNA methylation and sequence differences among regenerants were higher in the verification trials than in the optimisation trials. This also was observed in cluster analysis, where methylation-related markers showed 56% similarity among regenerants, and sequence-related markers showed 95% similarity among regenerants derived in the M10, M12 and M13 trials. Independently of marker type, the M13 regenerants exhibited higher variation than the M12 regenerants. In general, the regenerants in the respective trials grouped in the same clusters and the donors (used as controls of variation) grouped in a separate cluster. The increased variation levels among regenerants representing the M10, M12 and M13 trials is consistent with the AMOVA results, identifying up to 20.6% and 25.6% of variants for site DNA methylation alterations and SV, respectively. Our data support the notion (Machczyńska et al. 2014b) that methylation changes are less common in triticale tissue cultures than those related to mutations.

Analysis of Z_TTCIV characteristics in the verification experiment indicated that the respective values were higher than those in the optimisation experiment. This also was observed under control conditions. We think that the data may reflect fluctuations in the level of in vitro tissue culture-induced variation that may vary from experiment to experiment. Due to the long duration of tissue culture experiments and problems with obtaining sufficient numbers of regenerants, it was challenging to perform enough experimental repetitions to enable estimation of the experimental error, even though we were using the Taguchi method. AMOVA failed to demonstrate that the M10, M12 and M13 trials differed. However, when quantitative Z_TTCIV characteristics of the M12 and M13 trials were compared with the M10 control conditions, Z_DMV_CN, Z_DNMV_CN and Z_dMET_CN did discriminate between the M10–M12 and M10–M13 trials, suggesting that subtle differences in methylation context (symmetric and asymmetric sites) may differentiate control conditions from those used for plant regeneration with minimal differences compared with the donor.

To test the possibility that methylation context may be necessary for differentiating verification trials, we performed metAFLP analysis based on markers amplified using selective primers directed toward such sequences. Some primers identified site methylation changes affecting asymmetric sequences, whereas other primers detected differences involving both asymmetric and symmetric sites. The only methylation change that could be calculated univocally was the asymmetric methylation. In the case of CXG and CG contexts, it is assumed that markers related to CXG and CG sites may reflect both asymmetric and symmetric methylation changes. As the CXX is quantified univocally, one may extract asymmetric from symmetric methylation, thereby enabling quantification based on observed ‘events’ However, the input of asymmetric methylation change affecting CG and CXG sequences seems to be negligible. Thus, we decided not to complicate the formulae with additional corrections, and applied the formulae described in our previous studies (Machczyńska et al. 2014b) with small modifications concerning the denominator described earlier (Methods). If our reasoning is correct that specific selective primers should amplify markers related to distinct sequence contexts and reflect distinct aspects of plant DNA methylation, then the differences among contexts should not be random. Our hypothesis was consistent with ANOVA results, demonstrating that the differences among contexts were significant in the case of Z_DNMV_CN and Z_DMV_CN. In total, the CG and CXG contexts were the most affected by tissue culture; the CXX context was at comparable levels in Z_DNMV_CN and Z_DMV_CN, respectively. Our data is partly inconsistent with data presented for in vitro-derived tomato regenerants (Yang et al. 2018), where no changes in CG context were detected between regenerated plants and inbred lines, whereas CXG and CXX sequences were affected by demethylation (Yang et al. 2018). Congruency was evidenced for sugar beet, where differences in symmetric and asymmetric methylation levels of sugar beet leaves and callus contexts were noted. Methylation was higher in callus compared with leaves, and the most methylated sequences were CG context and CXG context, whereas CXX sequences essentially lack methylation (Zakrzewski et al. 2017). Recent bisulfite sequencing of some maize genome regions (Abbasi et al. 2019) revealed that de novo methylation of CG sequence prevailed over methylation of CXG sequence, and the CXX sequence had the lowest methylation levels. Our results showed that de novo methylation was higher than demethylation in the CXX sequence, which is consistent with the results for the control (M10) trial but not for the M13 trial that verified maximum variation.

Methylation changes affecting asymmetric methylation are regulated by epigenetic mechanisms (Cao and Jacobsen 2002; González et al. 2011) and are usually infrequent (Meyer 2011). Conversely, the increased levels of changes within the CG and CXG sequences suggest that different mechanisms are responsible for their preservation and origin (Meyer 2011). The results demonstrated that Z_DMV_CN was relevant within CG and CXX contexts in differentiating M12 vs. M13 and M10 vs. M13 trials. Z_DNMV_CN was relevant within CXG and CXX methylation contexts in differentiating M10 vs. M12 trials. Detailed analysis of DNA methylation changes showed that they have essential roles in tissue cultures, and Z_DMV_CN with Z_DNMV_CN are the variations that influence maximum and minimum numbers of differences between donor and anther-derived regenerant plants in barley tissue culture. Our study indirectly supports the notion that metAFLP analysis is sensitive enough to detect DNA methylation differences in distinct sequence contexts that occur during in vitro tissue culture.

One of the hypotheses of tissue culture-induced variation considers that sequence changes may be due to DNA demethylation and activation of mobile elements (Azman et al. 2014; Orłowska et al. 2016; Xu et al. 2004). Our data indicate that DNA methylation/demethylation in barley affects symmetric sites and depends on the trial. However, Z_SV_CN was not correlated with methylation change when all metAFLP markers were used for the analysis, and no correlation within contexts was detected when trials were not assumed. Lack of such correlation suggests that the verification trial results are averaged; thus statistics cannot detect strong correlations. This notion is supported by the fact that a number of correlations were identified when contexts within trials were analysed. The differences in correlation coefficients between Z_SV_CN and Z_dMET_CN, Z_DMV_CN and Z_DNMV_CN within a given context and for the given trial demonstrate that tissue culture conditions influence different contexts. Under control conditions, Z_SV_CN was negatively correlated with Z_dMET_CN but positively correlated with M13. The same evidence was observed in the CXX context, suggesting that changing in vitro tissue culture plant regeneration conditions influences the relationship between SV and DNA methylation pattern, and these changes are responsible for increased variation in the M13 trial compared with the M10 trial.

Culture conditions that minimise variations among regenerated plants did not exhibit correlations between Z_SV_CN and other methylation-related metAFLP characteristics. Our data suggest that, under control conditions, Z_SV_CN is due to DNA methylation changes; however, in the CG and CXG contexts of the M10 and M13 trials, Z_DNMV_CN was higher than Z_DMV_CN. This change is not consistent with the transposable elements (TE s) activation hypothesis. Conversely, the opposite was observed for the CXX context in the M13 trial. It is not clear whether these changes in the asymmetric context are really correlated with TE activity. We speculate that changing in vitro tissue culture conditions may influence Z_SV_CN via alterations in DNA methylation patterns (both Z_DMV_CN and Z_DNMV_CN).

Our results demonstrate that manipulating media component concentrations and time spent in tissue culture induction and regeneration media may influence the in vitro tissue culture-induced variation levels. We showed that the number of (epi)genetic differences between donor and regenerant plants can be increased or decreased by modifying some culture components and parameters. Finally, the metAFLP characteristics describing methylation changes in different methylation contexts enabled the identification of subtle methylation ‘events’ affecting the plant genome during in vitro plant regeneration.

## Electronic supplementary material

Below is the link to the electronic supplementary material.

Electronic supplementary material 1 (PDF 519 kb)

Electronic supplementary material 2 (XLSX 70 kb)

Electronic supplementary material 3 (PDF 122 kb)
