# Supplementary material for: Precise evaluation of tissue culture-induced variation during optimisation of in vitro regeneration regime in barley
Source: Plant Mol Biol. 2020 Feb 11;103(1):33–50. doi: 10.1007/s11103-020-00973-5 (PMC7170832; doi:10.1007/s11103-020-00973-5)
Supplement: Supplementary file 1 — Electronic supplementary material 1 (PDF 519 kb) [file 11103_2020_973_MOESM1_ESM.pdf]

Precise evaluation of tissue culture-induced variation during optimisation *in vitro* regeneration regime in barley

Plant Molecular Biology

Renata Orłowska, Piotr T. Bednarek*
Plant Breeding and Acclimatization Institute–National Research Institute, Department of Plant Physiology and Biochemistry, 05-870 Błonie, Radzików, Poland.
*corresponding author: p.bednarek@ihar.edu.pl

Online Resource

Table S1 Oligonucleotides applied for metAFLP in barley studies

metAFLP oligomer	Sequence 5'→3'
**Adaptors**	
**Ad1 *Acc65*I**	CTC GTA GCA TGC GTA CA
**Ad2 *Acc65*I**	GTA CTGTACGCATGCTAC
**Ad1 *Kpn*I**	CTC GTA GCA TGC GTA CAG TAC
**Ad2 *Kpn*I**	TGTACGCATGCTAC
**Ad1 *Mse*I**	TAC TCA GGA CTC ATC
**Ad2 M*se*I**	GAC GAT GAG TCC TGA G
**Preselective primers**	GAT GAG TCC TGA GTA AC
**Presel *Acc56*I/*Kpn*I**	GCA TGC GTA CAG TAC C
**Presel *Mse*I**	GAT GAG TCC TGA GTA AC
**Labeled ^{32}P selective oligonucleotides**	
**CpG-GAC**	CA TGC GTA CAG TAC CGA C
**CpG-GCA**	CA TGC GTA CAG TAC CGC A
**CpG-GGC**	CA TGC GTA CAG TAC CGG C
**CpG-TCG**	CA TGC GTA CAG TAC CTC G
**CpXpG-AGA**	CA TGC GTA CAG TAC CAG A
**CpXpG-AGC**	CA TGC GTA CAG TAC CAG C
**CpXpG-AGG**	CA TGC GTA CAG TAC CAG G
**CpXpG-ATG**	CA TGC GTA CAG TAC CAT G
**CpXpG-TGC**	CA TGC GTA CAG TAC CTG C
**CpXpG-TTG**	CA TGC GTA CAG TAC CTT G
**CXX-ATT**	CA TGC GTA CAG TAC CAT T
**CXX-TAA**	CA TGC GTA CAG TAC CTA A
**Selective oligonucleotides**	
**M-CAC**	GAT GAG TCC TGA GTA ACA C

**M-CGT**	GAT GAG TCC TGA GTA ACG T
**M-CTA**	GAT GAG TCC TGA GTA ACT A

Table S2 Primers used in metAFLP for donors, optimisation and verification experiments

	**Plant material**	
**Primers combinations**	Donors	Regenerants
**CpG-GAC/MCGT**	1	1
**CpG-GCA/MCGT**	1	**-**
**CpG-GGC/MCAC**	1	1
**CpG-TCG/MCAC**	1	**-**
**CpXpG-AGA/MCGT**	1	1
**CpXpG-AGC/MCAC**	1	1
**CpXpG-AGG/MCGT**	1	1
**CpXpG-ATG/MCGT**	1	**-**
**CpXpG-TGC/MCGT**	1	**-**
**CpXpG-TTG/MCAC**	1	**-**
**CpXpG-TTG/MCGT**	1	1
**CXX-ATT/MCAC**	1	1
**CXX-TAA/MCGT**	1	1
**Total**	13	8

Table S3 Percentage of polymorphic loci (*%P*) and Shannon's information index (*I*) for metAFLPs amplified from DNA of anther-derived regenerants used in Experiment 1 (M1–M9 trials)

	MetAFLP platform			
Trial	*Acc65*I/*Mse*I-*Kpn*I/*Mse*I (M)		*Kpn*I/*Mse*I (K)	
	%P	*I*	*%P*	*I*
M1	2.7	0.013	2.21	0.011
M2	1.23	0.006	0.98	0.005
M3	3.44	0.013	3.69	0.014
M4	0	0	0.49	0.003
M5	2.46	0.013	2.21	0.012
M6	1.47	0.008	0.25	0.002
M7	1.97	0.008	2.95	0.014
M8	4.42	0.022	3.19	0.017
M9	6.39	0.032	2.95	0.012
Mean	2.68	0.013	2.1	0.01

M1, control experiment; M2–M9, trials required by the Taguchi method for optimisation of three factors at three levels. *Acc65*I/*Mse*I-*Kpn*I/*Mse*I (M) platform, markers related to DNA methylation change; *Kpn*I/*Mse*I (K) platform, markers related to DNA sequence mutation.

Table S4 The number of events evaluated for each trial. Z states for all marker contexts taken together

**Events**	**Trials**								
	**M1**	**M2**	**M3**	**M4**	**M5**	**M6**	**M7**	**M8**	**M9**
**Z_0000**	138	146	128	90	136	150	139	130	126
**Z_0001**	4	1	9	2	0	3	2	92	4
**Z_0010**	5	5	5	3	5	5	5	0	5
**Z_0011**	88	88	88	54	87	96	87	0	87
**Z_0100**	19	25	23	13	21	21	16	33	30
**Z_0101**	19	8	20	3	23	6	23	50	20
**Z_0110**	10	10	10	6	10	10	10	0	9
**Z_0111**	22	22	22	12	23	14	23	0	24
**Z_1000**	10	10	8	6	10	10	10	0	11
**Z_1001**	10	10	10	6	10	10	10	0	10
**Z_1010**	0	2	4	1	2	5	3	14	5
**Z_1011**	6	6	5	3	6	5	6	16	5
**Z_1100**	100	100	102	60	99	100	100	0	98
**Z_1101**	5	5	5	3	6	5	5	0	6
**Z_1110**	11	10	11	6	12	10	10	109	10
**Z_1111**	1588	1587	1585	953	1585	1585	1586	1591	1585

Table S5 The arrangement of analysis for the presence of outliers within each of the M1-M9 trials based on TTCIV values.

**Trial**	**Regenerant**	**Grubbs Test**	
		Z-score	p-value
**M1**	1	1.332	0.745
	2	-0.065	
	3	-1.115	
	4	-0.772	
	5	0.620	
**M2**	1	0.152	0.631
	2	0.921	
	3	-1.381	
	4	0.921	
	5	-0.613	
**M3**	1	-1.073	1.000

	2	0.387	
	3	0.899	
	4	-1.073	
	5	0.860	
**M4**	1	1.155	< 0.0001
	2	-0.577	
	3	-0.577	
**M5**	1	-0.894	1.000
	2	0.104	
	3	1.088	
	4	0.842	
	5	-1.139	
**M6**	1	0.238	0.510
	2	1.436	
	3	-0.955	
	4	0.238	
	5	-0.955	
**M7**	1	1.533	0.318
	2	-0.538	
	3	0.497	
	4	-0.746	
	5	-0.746	
**M8**	1	0.604	1.000
		0.325	
	3	1.160	
	4	-1.064	
	5	-1.026	
**M9**	1	0.809	0.720
	2	-0.717	
	3	-0.717	
	4	-0.717	
	5	1.342	

TTCIV – Total Tissue Culture-Induced Variation

Table S6 Percentage of polymorphic loci (*%P*) and Shannon's information indices (*I*) for the metAFLP markers (M and K platforms) identified in the M10–M13 trials

Trial	metAFLP marker			
	*Acc65*I/*Mse*I-*Kpn*I/*Mse*I (M)		*Kpn*I/*Mse*I (K)	
	%P	*I*	*%P*	*I*

M10	7.84	0.043	4.58	0.025
M12	9.48	0.050	4.25	0.02
M13	12.42	0.062	8.50	0.045
Mean	9.91	0.052	5.77	0.030

Table S7 The number of events evaluated for each trial in Experiment 2. Z states for all sequence contexts taken together

**Events**	**Trials**		
	**M10**	**M12**	**M13**
**Z_0000**	8	12	18
**Z_0001**	2	5	0
**Z_0010**	0	1	2
**Z_0011**	39	51	83
**Z_0100**	9	9	6
**Z_0101**	2	2	1
**Z_0110**	1	1	0
**Z_0111**	11	10	17
**Z_1000**	7	6	24
**Z_1001**	2	1	3
**Z_1010**	6	7	13
**Z_1011**	18	18	34
**Z_1100**	28	35	68
**Z_1101**	9	9	26
**Z_1110**	7	4	10
**Z_1111**	467	543	923
