# Supplementary material for: Precise evaluation of tissue culture-induced variation during optimisation of in vitro regeneration regime in barley
Source: Plant Mol Biol. 2020 Feb 11;103(1):33–50. doi: 10.1007/s11103-020-00973-5 (PMC7170832; doi:10.1007/s11103-020-00973-5)
Supplement: Supplementary file 3 — Electronic supplementary material 3 (PDF 122 kb) [file 11103_2020_973_MOESM3_ESM.pdf]

Precise evaluation of tissue culture-induced variation during optimisation *in vitro* regeneration regime in barley

Plant Molecular Biology

Renata Orłowska, Piotr T. Bednarek*
Plant Breeding and Acclimatization Institute–National Research Institute, Department of Plant Physiology and Biochemistry, 05-870 Błonie, Radzików, Poland.
*corresponding author: p.bednarek@ihar.edu.pl

ESM_2

Description of completing the calculator sheet for 'RELEASE-CALCULATOR-SIMPLIFIED-EXAMPLE'

1. In the 'RAW DATA' sheet, markers / DNA fragments obtained from the digestion of DNA for the "D" donor plant and the "R1" regenerant with *Acc65*I / *Mse*I enzymes should be entered. Similarly, fill in the DNA data for the donor plant 'D' and the regenerant 'R1' digested with *Kpn*I / *Mse*I enzymes.
   It is important to separately analyze markers / DNA fragments derived from amplification with individual primers corresponding to CXX, CG, and CXG methylation contexts.
   In the presented calculator, calculations can be made for the donor plant, and one regenerate at the same time. If there are more regenerants in the analysis, the data for each regenerant is counted separately. And then, the data are transferred to the appropriate sheets 'CXX,' 'CG,' and 'CXG' as the following items: R1, R2, R3, etc.
2. The sum for individual events is calculated automatically in the 'EVENTS' sheet. Calculation of the number of 'events' for individual methylation contexts is done in the same way and can be carried out in the same 'RAW DATA' sheet. It is important that the sheets named 'CXX,' 'CG,' and 'CXG' receive data corresponding to specific methylation contexts
3. The sum of 'events' for data from the CXX methylation context should be inserted into the 'CXX' sheet, data on the CG and CXG context should be inserted into the appropriate 'CG' and 'CXG' sheets. In sheets 'CXX,' 'CG' and 'CXG' data can be counted for an unlimited number of regenerants. Formulas from R1 can be extended to more regenerants.

4. The ‘TOTAL’ sheet contains qualitative and quantitative data for the metAFLP characteristics in general and in methylation contexts, which are the basis for estimating the sequence and methylation changes in the tested plant materials derived from in vitro cultures.
